# European Preparedness for Japanese Encephalitis Virus Through Alignment of Animal Health Laboratory Diagnosis

**DOI:** 10.1155/tbed/5516160

**Published:** 2025-06-10

**Authors:** Karen L. Mansfield, Insiyah Parekh, Thomas Bruun Rasmussen, Louise Lohse, Ann Sofie Olesen, Nolwenn M. Dheilly, Gaëlle Gonzalez, Camille Victoire Migné, Mathilde Gondard, Teheipuaura Helle, Tobias Lilja, Johanna F. Lindahl, Wim H. M. van der Poel, Frank Harders, Gebbiena M. Bron, Melle Holwerda

**Affiliations:** ^1^Department of Virology, Vector-Borne Diseases Group, Animal and Plant Health Agency (APHA), Addlestone, Surrey, UK; ^2^Department of Virology and Microbiological Preparedness, Statens Serum Institut (SSI), Copenhagen, Denmark; ^3^Virology Department, Animal Health Laboratory, ANSES, Maisons-Alfort, France; ^4^Pathogen Discovery Laboratory, Institut Pasteur, Paris, France; ^5^Department of Microbiology, Swedish Veterinary Agency (SVA), Uppsala, Sweden; ^6^Department of Animal Health and Antibiotic Strategies, Swedish Veterinary Agency (SVA), Uppsala, Sweden; ^7^Department of Virology and Molecular Biology, Wageningen Bioveterinary Research—WUR (WBVR), Lelystad, the Netherlands; ^8^Department of Animal Health, Animal Welfare and Food Safety, Norwegian Veterinary Institute (NVI), Ås, Norway

**Keywords:** diagnostics, emerging, Japanese encephalitis, orthoflavivirus, zoonosis

## Abstract

Outbreaks of Japanese encephalitis (JE) can have severe health and economic impacts in both humans and susceptible animal species and are estimated to cause ~68,000 human disease cases in Asia annually. The disease is caused by infection with the mosquito-borne JE virus (JEV), which continues to expand its geographical range from its endemic region in Asia. Since appropriate vertebrate host and mosquito vector species are present in Europe and average European summer temperatures continue to increase, JEV introduction could lead to the establishment of the pathogen in native mosquito species and wild birds and disease outbreaks among humans, pigs, and horses. Incursions could occur through movements of infected pigs and mosquitoes but also via migratory birds that act as reservoirs. Introduction and establishment of JEV in these populations may not be apparent at first, providing time for virus spread before spillover to the human population. Further complicating serological detection of JEV is the extensive cross-reactivity with other orthoflaviviruses circulating in Europe (i.e., tick-borne encephalitis virus [TBEV], West Nile virus [WNV], and Usutu virus [USUV]). In addition, viremia in clinical cases may be short, hindering virus detection. To facilitate European preparedness for detection, surveillance, and monitoring of JEV introduction and spread, five veterinary national reference laboratories in Europe collaborated with the aim to align JEV diagnostic pipelines to prepare for future emergence of JEV in Europe. All institutes assessed established and newly developed serological and molecular assays to build capability with sensitive and specific diagnostic tools for JEV detection. Additionally, methods for whole-genome sequencing (WGS) were established and compared. In summary, this project provides a framework for communication and international collaboration between arboviral researchers at national veterinary institutes. The sharing of knowledge and expertise, and alignment of diagnostic techniques, has facilitated improvement of diagnostic pipelines for JEV detection and contributed to preparedness for JEV introduction into Europe.

## 1. Introduction

The mosquito-borne orthoflavivirus, Japanese encephalitis (JE) virus (JEV), is endemic throughout Asia, the western Pacific region, and parts of the Indian subcontinent [1], existing in enzootic cycles involving *Culex* mosquitoes, vertebrate hosts, and wild avian reservoir species [2, 3]. The primary vector for enzootic maintenance within amplifying hosts (waterbirds and swine), along with spillover to humans and horses, is *Culex* (*Cx*.) *tritaeniorhynchus* [2, 3], although other mosquito species can vector JEV. Genetic analysis of the viral genome suggests that strains of JEV can be phylogenetically differentiated into five genotypes (genotype I [G1], genotype II [GII], genotype III [GIII], genotype IV [GIV], and genotype V [GV]) associated with different geographical distributions [4–6]. Analysis of whole-genome sequence has revealed 11.4%–19.6% nucleotide divergence and 2.1%–6.5% amino acid divergence among GI–GIV, where GIV showed the greatest amino acid divergence [7]. The more recently emerged GV appears to be even more divergent from the other genotypes, with divergence of 20.3%–21.4% (nucleotide) and 8.4%–10% (amino acid) [8]. In recent years, JEV has spread into new areas, including the incursion and expansion of GIV in Australia [9], where until 2021 incursions of JEV had only been detected in northeast Queensland. However, in 2021 and 2022, JEV was reported from the Tiwi Islands of the Northern Territory and southeastern Australia, respectively. Human cases were reported from both regions, and several pig farms were affected [9], with some piggeries reporting a stillborn rate of up to 70% [10]. The geographical range of JEV is at risk of further expansion due to global movement of animals and animal products, which could potentially be infected with JEV, changes in land use which may facilitate the expansion of mosquito populations, and the impact of climate change on mosquito population dynamics, leading to the distribution of mosquito vectors and JEV expansion into previously non-endemic areas [11]. Human infection with JEV can lead to JE, the principal cause of viral encephalitis cases in Asia causing ~68,000 human cases annually, predominantly in children below 15 years of age [1]. While most infections are asymptomatic, clinical symptoms vary from headache and nonspecific febrile illness to seizures, flaccid paralysis, and cardiac arrest [12]. The case fatality rate is 5%−50% of symptomatic infections, again with the highest rates observed in children below 15 years of age and in the absence of vaccination targeting this age group [12, 13]. Up to 50% of survivors can suffer from permanent neurological sequelae [14]. Some other mammals are also susceptible to JEV infection, with gestational complications such as stillbirth and abortion in pigs and neurological disease in horses [5, 15, 16]. While horses and humans are dead-end hosts for JEV [5], pigs are an amplifying host due to a transient high titer viremia shortly after infection [17]. Hence, in regions with high-density pig production, there is an increased risk of transmission to humans, particularly in the presence of competent mosquito vectors [16, 18]. Furthermore, antibodies against JEV have been detected in poultry [19], and modeling suggests that poultry may act as a reservoir in endemic areas, enabling JEV to become established in the absence of pigs and increasing the risk of transmission to humans in close proximity to poultry [20]. Alongside the potentially high economic impact of a JEV outbreak in pigs, this highlights that rapid detection and control of JEV in domestic animals would support the reduction of JEV transmission to humans (and other animals). There are human vaccines available [6], and some countries in Asia also have licensed animal vaccines for use in pigs and horses [6].

Diagnosis of JEV infection in humans and animals can be challenging due to the occurrence of asymptomatic infections and nonspecific clinical manifestations. Antemortem diagnosis is usually through antibody detection in serum or cerebrospinal fluid [21], using IgM- and IgG-isotype ELISAs as an indicator of acute or recent infection (IgM) or past exposure or vaccination (IgG), respectively. However, conventional ELISA methods are unable to differentiate between antibodies induced by infection and those induced by vaccination [21, 22]. Only one serotype of JEV has been identified [21]; however, orthoflaviviruses cross-react serologically, hence prior exposure to other orthoflaviviruses (e.g., West Nile virus [WNV]) leads to ELISA cross-reactivity which can further complicate diagnosis [21, 23]. Antemortem diagnosis can also be established through the confirmation of viremia via detection of viral antigens or viral genomes in serum using plaque assays and molecular assays, respectively [21], although this can also be problematic due to the short duration of viremia in JEV infections.

In Europe, suitable vertebrate hosts and mosquito vector species for JEV are already present [24], and average European summer temperatures continue to increase [25]. Hence, an incursion of JEV could lead to virus establishment in native mosquito species and wild birds, with spillover into humans, horses, domestic pigs, and potentially wild boar (*Sus scrofa*) [26]. Crucially, the introduction and establishment of JEV may not be apparent at first, which would facilitate virus spread before an index case (human or animal) is detected, as seen during the outbreak of GIV in Australia [9]. It is noteworthy that a study from July 2022 in New South Wales, Australia, reported JEV antibodies in people from areas where JEV-positive mosquitoes had been found, suggesting large-scale spread of the virus prior to detection [27]. To prepare for future emergence of JEV in Europe, there is a need for sensitive and specific diagnostic tools that could be applied to surveillance activities and laboratory diagnosis of clinical suspicions, enabling rapid detection so that virus incursions can be identified and controlled. Due to the increasing arbovirus activity in Europe [28] and recent JEV outbreaks in Australia [9], a collaboration was established in 2022 under the umbrella of the Collaborating Veterinary Laboratories (CoVetLab, www.covetlab.org) between national veterinary institutes from five European countries: Animal and Plant Health Authority (APHA), United Kingdom; ANSES, France; Statens Serum Institut (SSI), Denmark; Swedish Veterinary Agency (SVA), Sweden; and Wageningen Bioveterinary Research (WBVR), the Netherlands. The overarching aim was to facilitate preparedness for an incursion of JEV into Europe through the alignment of diagnostic pipelines, with objectives to (1) summarize the current understanding on host and vector parameters for transmission models in Europe, (2) identify appropriate pipelines for serological detection of exposure to JEV, and (3) identify appropriate pipelines for rapid virus detection and genotyping. In the event of any future incursion of JEV into Europe, this study will have enhanced European preparedness for detection, surveillance, and monitoring for JEV introduction, to ultimately support virus control strategies.

## 2. Materials and Methods

### 2.1. Samples for Assessment of Diagnostic Assays

Samples for initial ELISA comparison (Section 2.2) comprised of sera (*n* = 12) from JEV-positive pigs (kindly provided by the World Organization for Animal Health [WOAH] Reference Laboratory for JEV, South Korea). For interlaboratory comparison, this project used the 2023 equine encephalitis proficiency panel samples distributed by the EU Reference Laboratory (EURL) for Equine Diseases (ANSES, Maisons-Alfort, France). Although the intended purpose of the proficiency panel was WNV diagnosis, it contained several blinded JEV samples for molecular and serological testing and therefore provided a useful assessment of JEV testing pipelines at each participating laboratory. The proficiency panels comprised experimentally infected horse sera for serology (*n* = 16) and spiked tissue homogenates for molecular analysis (*n* = 19), with blinded panels comprised of samples that were positive for viruses including WNV, Usutu virus (USUV), tick-borne encephalitis virus (TBEV), and JEV. This included serum samples (*n* = 3) from horses experimentally infected with JEV Tottori strain (GenBank accession: AB594829). The three blinded samples in the panel for molecular analysis were derived from dilution of the strains JEV strain Tottori and JEV Nakayama, with starting titers of 6.3 × 10^5^ plaque-forming unit (PFU)/mL and 5.1 × 10^5^ PFU/mL, respectively.

### 2.2. Established Protocols for Serological Detection of JEV-Specific Antibodies at Partner Institutes

In the absence of a commercially available JEV-specific ELISA, two ELISAs were initially compared for cross-reactivity using sera from infected pigs (detailed in Section 2.1): the ID Screen Flavivirus Competition ELISA (Innovative Diagnostics) for detection of anti-pr-E antibodies and the ID Screen West Nile IgM Antibody Capture ELISA (Innovative Diagnostics). For interlaboratory comparison, virus neutralization assays were also assessed. The plaque reduction neutralization test (PRNT) was undertaken on Vero C1008 cells using a GI strain of JEV (UVE/JEV/2009/LA/CNS769 [29]) and standard techniques as previously described [30]. Virus neutralization test (VNT) was also applied to differentiate neutralizing antibodies against WNV (lineage 1 Israel 1998), USUV (France Africa 3 strain from 2018), and JEV Nagayama with initial titer at 100 TCID_50_/50 µL as previously described [31]. Using this method, a serum sample was considered positive if no cytopathic effect was observed at a 1:20 dilution. Due to cross-reactivity between orthoflaviviruses, JEV-specific antibodies were confirmed in samples with the highest neutralization capacity against JEV and a fourfold difference in neutralization titer.

### 2.3. Established Protocols for Rapid Molecular Detection of JEV

For rapid molecular detection of JEV, four different JEV-specific probe-based RT-PCR protocols were in use between the partner institutes, along with one pan-orthoflavivirus RT-PCR (Table 1, Table S1).

### 2.4. Genotyping Through Whole-Genome Sequencing (WGS)

Two methodologies were assessed and compared for WGS of two JEV strains, the prototype GIII Nakayama strain (GenBank accession: EF571853 and HE861351) and GI Tottori (GenBank accession: AB594829).

#### 2.4.1. Amplicon-Based WGS for JEV

Two different primer sets against JEV GI and GIII were designed by two participating laboratories (SVA and ANSES) using the Primal Scheme design tool to produce 400-bp overlapping amplicons (Table S2). Nucleic acid samples were extracted using the ID Gene Mag Universal Extraction MAG 384 kit (Innovative Diagnostics), and reverse transcription was performed using the SuperScript IV VILO Master Mix that includes random hexamers, according to manufacturer instructions (ThermoFisher Scientific). A multiplex, tiled PCR method for targeted enrichment and WGS of JEV GI and GIII was developed using Oxford nanopore technology as previously described [37]. Two primer pools were prepared with alternating primer sets, and targeted amplifications were undertaken using the Q5 High-Fidelity 2X Master Mix kit according to manufacturer instructions (New England Biolabs). PCR products were pooled appropriately and purified using the HighPrep PCR Clean-up System (MagBio Genomics Inc., USA). Sequencing libraries were prepared using the Native Barcoding Genomic DNA protocol with the NBD114.24 Native Barcoding kit (Oxford Nanopore Technologies, UK) and loaded onto a FLO-MIN114 R10.4.1 flowcell (Oxford Nanopore Technologies, UK). A 72-h run was conducted with standard settings using MinKNOW software (version 23.07.8). Raw reads were base called and demultiplexed using GUPPY (version 7.0.8) and the “highly accurate” model. Finally, genomic sequences were produced using a custom mapping workflow in Geneious Prime (version 2022.0.2).

#### 2.4.2. Shotgun WGS for JEV

A shotgun protocol was developed for WGS of JEV GI and GIII, based on a previously published nontargeted metagenomics approach [38]. Nucleic acid samples were treated with the DNA-free DNA Removal kit (Thermo Fisher Scientific) and double-stranded cDNA generated using the SuperScript III First-Strand Synthesis kit (Invitrogen) and the NEBNext Ultra II Non-Directional RNA Second Strand Synthesis kit (New England Biolabs). Libraries were generated using the Nextera XT DNA Library Preparation Kit (Illumina) and run on the MiSeq platform using the Reagent Kit v2 300 bp (Illumina). Raw data reads were mapped using Geneious Prime version 2022.1.1. to JEV reference sequences (JEV/eq/Tottori/2003, GenBank accession AB594829 and Nakayama/MY/2009/P578662, GenBank accession HE861351).

## 3. Results and Discussion

### 3.1. An Overview on JEV Vectors and Hosts and Transmission Potential in Europe

#### 3.1.1. Mosquito Vectors for JEV

Transmission of JEV in endemic regions of Asia is dependent on mosquito species associated with rice paddy fields, in particular *Cx. tritaeniorhynchus* [39], while in southern Australia, where JEV was introduced in recent years, *Cx. sitiens* and *Cx. annulirostris* were predicted to be the most important vectors [40]. However, other *Culex* mosquito species (and mosquito genera) are competent vectors for JEV in endemic settings [41]. Several (*n* = 14) species are confirmed vectors for JEV, since infected field samples from endemic regions have been reported, and the species have been experimentally assessed for viral transmission capability (vector competence). These species include *Aedes* (*Ae*.) *albopictus*, *Ae. vexans*, *Ae. vigilax*, *Armigeres subalbatus*, *Cx. annulirostris*, *Cx. bitaeniorhynchus*, *Cx. fuscocephala*, *Cx. gelidus*, *Cx. pipiens*, *Cx. pseudovishnui*, *Cx. quinquefasciatus*, *Cx. sitiens*, *Cx. tritaeniorhynchus*, and *Cx. vishnui* (reviewed in Auerswald et al. [42]). Additional species (*n* = 11), although not confirmed through detection in field samples, have been experimentally shown to be competent vectors, *Ae. detritus*, *Ae. dorsalis*, *Ae. japonicus*, *Ae. kochi*, *Ae. nigromaculis*, *Ae. notoscriptus*, *Anopheles tessellatus*, *Cx. tarsalis*, *Culiseta* (*Cs*.) *annulata*, *Cs. inornata*, and *Verrallina funerea* (reviewed in Auerswald et al. [42]). Lastly, there are species (*n* = 26) from five mosquito genera where JEV was detected and/or isolated from field-caught mosquitoes, but where vector competence has not yet been investigated, including *Aedes* (four species), *Anopheles* (nine species), *Coquillettidia* (one species), *Culex* (seven species), and *Mansonia* (five species) mosquitoes (reviewed in Auerswald et al. [42]). There may also be varying vector competence for JEV in different populations of the same mosquito species, which is often unknown.

Although several important JEV mosquito vectors in Asia are absent in the vast majority of Europe, there are several that are present in Europe including *Ae. detritus*, *Ae. vexans*, *Cx. pipiens*, *Cx. tritaeniorhynchus*, and *Cs. annulata*. There are therefore several mosquito species in Europe that could be considered as potential vectors for JEV. Of these, the most likely European mosquito vector species for JEV is *Cx. tritaeniorhynchus* [43] for which transmission capability has been experimentally assessed [44]. However, *Cx. tritaeniorhynchus* is currently restricted to Greece, Albania, and Montenegro [45] and has relatively low abundance [46] compared to more widespread species such as *Cx. pipiens*. In mainland Europe, comparable assessments of the vector competence of native mosquito species for JEV are limited, although the highly abundant native *Cx. pipiens* in the United Kingdom and Belgium, along with native *An. plumbeus* in Belgium, have been shown to have transmission potential for JEV at 25°C [47,48–49]. Additionally, native *Cx. pipiens* and *Ae. albopictus* in France were shown to be competent vectors at 26°C [50]. In addition to native species, invasive species such as *Ae. japonicus* [51], which is expanding its geographical range north of the Alps [52], and *Ae. albopictus*, which is now established in many regions of continental Europe and geographically expanding [52], will further enhance the risk of JEV transmission in Europe. However, vector competence greatly depends on environmental temperatures, since experimentally infected *Cx. pipiens* from Belgium maintained at a 25/15°C day/night temperature gradient only yielded an 8% dissemination rate (detectable JEV in legs) and did not have detectable JEV in saliva at 14 days postinfection [49]. However, when maintained constantly at 25°C, 53.6% of exposed *Cx. pipiens* had a disseminated infection, and 13.3% had detectable virus in saliva, supporting transmission potential [49]. Therefore, it is evident that there are mosquito species throughout much of Europe that may constitute competent JEV vectors if the virus was to be introduced, with the risk of JEV transmission facilitated further through climatic changes such as increased temperature and rainfall.

#### 3.1.2. Vertebrate Hosts for JEV

**Figure 1:**
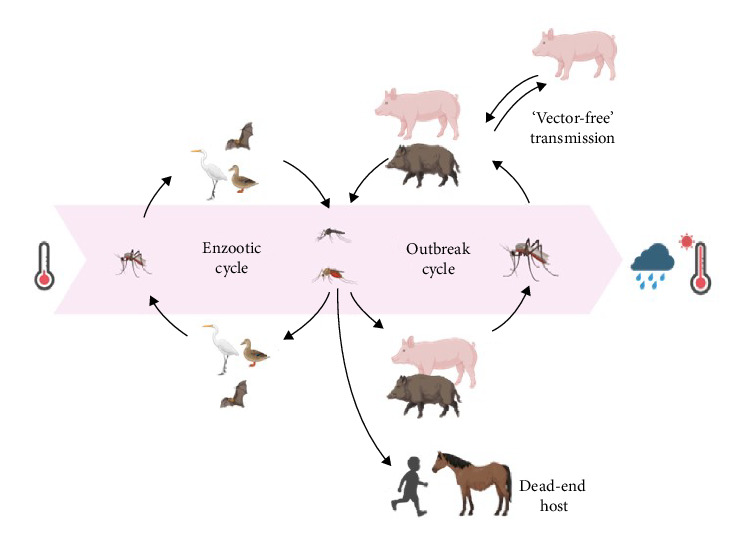
Transmission cycles of Japanese encephalitis virus in endemic and enzootic areas, highlighting the impact of climate changes (increased temperature and rainfall) on the risk of virus outbreaks, and the potential involvement of wild boar. Created with BioRender.com. Mansfield, K. (2025) https://BioRender.com/n6xdcp3.

In JEV-endemic areas, the vertebrate hosts of JEV are pigs and birds (Figure 1). Pigs are efficient amplifying hosts since they tend to develop a high titer viremia when infected, which can last up to 4–5 days [18]. Due to the high turnover rate in the pig industry, there are usually several susceptible pigs present in a population at any one time [53]. In addition to the susceptibility of pigs to mosquito-borne transmission, there is also evidence that “vector-free” transmission between pigs is possible, where pigs can shed sufficient JEV in their oronasal secretions to infect other pigs through direct transmission, further increasing the importance of the role of pigs in JEV transmission [18, 54].

JEV can also be present in boar semen, implicating a potential risk for sexual transmission [55, 56], including transmission via artificial insemination. More than 90 domestic and wild bird species have been found to be infected by JEV in endemic regions, with wading birds of the *Ardeidae* family, such as egrets (*Egretta garzetta*) and herons (*Nycticorax nycticorax*), particularly susceptible to infection, exhibiting high-titer viremia sustained for up to 5 days [57, 58]. These bird species are present in flooded fields of arable land used for growing semiaquatic crops such as rice (paddy fields), where large numbers of mosquitoes hatch, hence, they are an important source of infection for mosquitoes [59]. The migratory movement of these birds would therefore be an important risk factor for JEV introduction into Europe, particularly as there are rice paddy fields in countries across continental Europe, including France, Spain, Portugal, and Italy [60]. In Australia, the recent outbreaks and spread of JEV were thought to be dependent on ardeid birds, other waterfowl, and feral and domestic pigs. However, due to the large populations of feral pigs in parts of Australia, it is possible that JEV could become endemic in these areas in the event of further outbreaks. In addition to these hosts, some bat species have been shown to be infected by JEV and may potentially serve as reservoir hosts [9]. In Europe, it is likely that the same bird populations that are involved in the spread of other orthoflaviviruses such as WNV and USUV could act as hosts for JEV. The large-scale spread of JEV in Australia prior to detection in 2022, when cases were reported in large parts of the country [9], highlights that the virus can readily spread to new areas undetected. In a similar way, JEV may potentially spread for some time in Europe before detection through surveillance.

#### 3.1.3. Transmission Potential of JEV in Europe

In 2017, the European Food Standards Agency (EFSA) conducted a risk assessment for 36 vector-borne diseases, to determine the risk of introduction into Europe through movement of livestock and pets [28]. The semiquantitative assessment estimated that JEV would not be introduced into Europe more than 0.001 times per year, although the probability of establishment following an introduction was estimated to be high (probability of 0.1–1 per introduction) depending upon the region of Europe. However, sporadic introductions may be expected in Europe, as demonstrated by suspected JEV genome detection in Italian birds and *Cx. pipiens* [61]. This does not necessarily lead to establishment, even when suitable hosts (water birds and pigs) and vectors (*Culex* spp. mosquitoes) are present. However, specific conditions such as increased temperatures and rainfall/flooding (facilitating more standing water), changes in land use, and extensive pig farming, in association with the presence of competent mosquito vectors, may increase the risk of JEV becoming established [24, 62]. A similar scenario occurred in New York in 1999, where several factors such as the presence of large populations of susceptible humans and animals, appropriate mosquito vectors, and favorable environmental conditions came together, leading to a “perfect storm” and a WNV outbreak which subsequently spread across North America [63].

Apart from the risk assessment created by EFSA in 2017 [28], there are currently no comprehensive risk maps available for JEV. However, the EFSA JEV disease profile [64] is a valuable source of information, containing a regularly updated overview of the current and historic distribution of reported cases in a geographic range map, along with information on the current observational and experimental data. Mosquito vector ranges (native and invasive) are available from the European Centre for Disease Prevention and Control (ECDC) [52] and provide supporting information to inform assessment of potential risks for JEV transmission in specific regions of Europe. However, to facilitate living risk maps for the introduction and establishment of JEV and other exotic vector-borne viruses, it is necessary to collate not just regional data on activity and availability of mosquito vectors but also data on susceptibility (host competence) and abundance of livestock and wildlife (potential hosts). The risk of incursion into Europe is currently considered to be low; however, the likelihood of transmission after an incursion is expected to be high, due to environmental conditions in Europe that are suitable for virus circulation [24]. Although the most likely route of incursion into Europe would be via infected migratory birds, any subsequent spread would likely be due to the movement of infected mosquitoes, pigs, and wild birds [24]. Assessment of the potential routes of introduction into Europe, and associated likelihood and risk, should therefore also include the distribution of mosquito vectors, wild boar, and other vertebrate hosts, as well as the migratory routes of wild birds. The variation in these parameters between different countries in Europe, particularly the distribution of mosquito species and landing sites for migratory birds, suggests that the risk of JEV introduction and establishment will vary geographically. The distribution of key mosquito species varies throughout Europe; although *Cx. tritaeniorhynchus* are currently geographically restricted to the eastern Mediterranean region [45], other species with potential for transmission, such as *Cx. pipiens*, *Ae. detritus*, and *Ae. vexans*, are more widespread [52]. Similarly, although pig production is widespread throughout Europe, there are several regions with high-density pig production, including Denmark and the Netherlands [65]. In terms of wild boar, modeling has predicted greater population densities in central and southern European regions, although the true population density in Europe remains unclear [66]. Therefore, essential factors that could facilitate JEV transmission in Europe are present. However, major knowledge gaps remain on virus replication and onward transmission efficiency at different temperatures in local mosquito species, the transmission efficiency to mammalian species such as humans and pigs, and thereby the relevance of direct transmission from pig-to-pig. This further emphasizes the need to have serological and rapid detection methodologies (pipelines) for JEV in place.

### 3.2. Establishment of a Pipeline for Serological Detection of Anti-JEV Antibodies

In the first instance, two commercially available ELISA kits were compared for the detection of anti-JEV antibodies, including the potential for cross-reactivity with a WNV-specific IgM assay, using a panel of sera from infected pigs, as detailed in Section 2.1. Although some studies have reported cross-reactivity between IgM antibodies for different orthoflaviviruses [23], there was a lack of cross-reactivity observed with the ID Screen West Nile IgM Capture ELISA (Innovative Diagnostics) (negative cutoff, S/P ≤ 35%) (Table 2), confirming that this particular ELISA would not have utility for JEV serological diagnosis. However, this observation was not unexpected, since higher levels of orthoflavivirus antibody cross-reactivity have been reported for IgG-based serological assays, compared to IgM-based assays [23]. Additionally, this IgM ELISA kit has been validated for use with horse sera only, so may have limitations to detection of antibodies in pig sera. In comparison, the ID Screen Flavivirus Competition ELISA (Innovative Diagnostics) detected a measurable antibody response against JEV in all JEV-infected pig sera, where positive hemagglutination inhibition (HI) test titers (previously determined) aligned with positive ELISA results (positive cutoff S/N ≤ 40%) (Table 2). One benefit of the ID Screen Flavivirus Competition ELISA is the ability to detect anti-orthoflavivirus antibodies from a range of vertebrate species, including humans. However, the conserved nature of orthoflavivirus neutralizing antibody epitopes, located in domain III of the viral envelope protein, leads to antigenic cross-reactivity between orthoflaviviruses [67]. This tendency for orthoflavivirus cross-reactivity does not allow differentiation between antibodies raised by different orthoflaviviruses; hence, it is difficult to interpret ELISA results without the support of additional JEV-specific neutralization assays such PRNT or VNT. Since several orthoflaviviruses circulate in Europe (e.g., WNV, USUV, and TBEV), the competition ELISA only has utility as a primary screening tool prior to further confirmatory testing using a virus neutralization assay.

However, since orthoflavivirus neutralization assays are time consuming and labor-intensive and require CL-3 containment facilities, these limitations highlight the development need for an IgM-specific ELISA for specific and rapid serological detection of acute JEV infection for multiple species.

To test serological diagnosis pipelines within the consortium, the serology samples from the equine encephalitis EURL 2023 Proficiency panel (detailed in Section 2.1) were assessed (*n* = 16), which included three blinded JEV samples. Initial screening utilized the ID Screen Flavivirus Competition ELISA (Innovative Diagnostics, France); any positives or doubtful samples were then assessed by virus neutralization assay to differentiate between JEV-specific antibodies and antibodies raised against other orthoflaviviruses. All partner laboratories participated (except SVA) and were able to correctly detect the JEV samples within the EURL Proficiency Panel, with high sensitivity and 100% specificity (Table 3).

### 3.3. Establishment of a Standardized Molecular Pipeline for Virus Detection and Genotyping

#### 3.3.1. Established Protocols for Rapid Virus Detection

For molecular detection of JEV, several probe-based RT-PCR protocols were in use between the partner institutes (Table S1). To test the JEV molecular diagnostic pipelines, the panels of matched blinded samples provided to all participating laboratories for the annual EURL proficiency scheme for equine orthoflaviviruses (ANSES, France) were utilized, as detailed in the Materials and Methods (*n* = 19 tissue homogenates). All participating laboratories correctly detected the JEV samples within the proficiency panel (*n* = 3) using their probe-based molecular assay (Table 4), with all assays yielding Ct values that were similar or lower than the expected values provided by the EURL. Several laboratories used further assays to amplify larger amplicons for Sanger sequencing, including the pan-orthoflavivirus RT-PCR [36]. As expected, these data demonstrated some variability in Ct values obtained for the same blinded samples when tested at different institutes using different assays. Several of the assays yielded Ct values that were slightly higher than the expected Ct value provided by the EURL, although slight variation is to be expected. However, despite some interlaboratory variability, all assays assessed were able to rapidly detect JEV RNA correctly and within the thresholds of Ct value that would denote a positive detection. Hence, capability exists for both detection and genotyping, since Sanger sequencing on positive amplicons correctly generated through pan-orthoflavivirus RT-PCR identified all orthoflaviviruses in the panel, including identification of JEV. The full molecular report for the 2023 EURL Proficiency Panel is available on demand from adm.eurl-equinediseases@anses.fr.

#### 3.3.2. Genotyping Through WGS

The diagnostic pipelines can be extended further to include WGS, which would enable a more complete epidemiological analysis to be undertaken in the event of a JEV incursion. To enable JEV genotyping through WGS, a prerequisite of any bioinformatics pipeline for analysis of sequence data is an appropriate JEV reference genome. At the time of this study, 407 JEV whole genome sequences derived from a range of species were available on NCBI GenBank (Table S3).

Two methodologies for WGS were assessed (Section 2.4), including both shotgun nontargeted metagenomics and targeted amplicon-based methods for WGS of JEV Nakayama (GIII) and JEV Tottori (GI). Four sets of primers for amplicon-based sequencing protocols were assessed (two each for GI and GIII). The two primer sets designed by SVA enabled more extensive coverage of the JEV genome to include the 3′ and 5′ UTRs (primer sequences detailed in Table S4). In comparison, the primer pairs proposed by ANSES did not enable sequencing of the first amplicon (initiation codon ATG was omitted). A summary of results for amplicon-based sequencing of JEV GI and GIII is shown in Table 5. These data highlight that these primer sets are genotype-specific, since GIII-specific primers did not generate a consensus sequence from GI strains and vice versa.

In conclusion, the MinION amplicon sequencing method (AmpliSeq), performed with nanopore sequencing technology, enabled the detection and near complete genome sequencing of JEV-positive samples with Ct values up to 28 (equivalent to a viral load of ~580 genome copies). Additionally, the same samples were assessed using Illumina technology, and both RNA-seq and AmpliSeq approaches generated similar consensus sequences (data not shown). Shotgun sequencing (nontargeted metagenomics) was also able to detect and differentiate between JEV GI and GIII when applied to the JEV-positive samples included in the EURL Proficiency Panel (Table 6). However, this methodology yielded a lower number of mapped reads, when compared to amplicon-based sequencing. From these data, it is evident that both methodologies for WGS are effective for JEV detection, although the more targeted amplicon-based approach generated a greater number of mapped reads. If the ultimate aim is to recover the viral genome, then the effectiveness of the two approaches will depend upon the level of virus in the sample, since for samples with low viral load (high Ct values), AmpliSeq methodology often performs better.

There can also be cost benefits to AmpliSeq, since the increased specificity enables increased multiplexing and hence a reduction in the sequencing cost of each sample. However, when the viral genotype is unknown, or novel, the hypothesis-free shotgun sequencing approach could be advantageous. Additionally, since the primers reported here were designed against G1 and GIII only (reflecting the most prevalent genotypes in Asia [68]), their use is recommended to complement primer schemes for amplicon sequencing of other genotypes.

### 3.4. Alignment of Diagnostics for JEV and Future Perspective

Clinical diagnosis of infection with JEV in humans and animals remains a challenge. However, this project has facilitated preparedness for a JEV incursion into Europe, through a unique collaboration that aimed to align diagnostic pipelines for JEV in several European countries. Arthropod-borne viruses continue to pose a threat to human and animal health in Europe and worldwide, and this threat is further increased by rising global temperatures and extreme weather events such as flooding that affect mosquito population dynamics and increase the risk of virus transmission. This highlights the importance of collaborative working on a continental scale to enable preparedness for virus incursion and outbreak. The ability to utilize a robust diagnostic pipeline is crucial for detecting and controlling an introduction of JEV into Europe, including the ability to apply serological screening for vertebrate hosts and molecular testing of vertebrates and mosquitoes. Diagnostic testing using real-time RT-PCR, virus isolation, or virus neutralisation assays remain the gold standards for confirming JEV infection. Diagnosis through molecular detection of JEV RNA can be undertaken with postmortem tissues [21] as well as cerebrospinal fluids and blood samples, while the PRNT or VNT is the most specific serological method, particularly if the 90% threshold is applied [21]. For rapid throughput serology, there are currently no validated commercial JEV-specific ELISA kits available, and developing a JEV-specific diagnostic assay within the timeframe of the project would have been challenging. The development and validation of novel diagnostic tools, such as a JEV-specific ELISA, would require a diverse panel of positive serum samples from a variety of sources and of different JEV genotypes, to ensure robust demonstration of specificity. Therefore, this project relied on the sharing of knowledge and protocols between participating laboratories, to support JEV diagnosis.

JEV is detected throughout Southeast Asia [7, 69], and its distribution continues to expand in Asia and Australasia [59]. Interestingly, in 2016, a human coinfection with JEV and yellow fever virus was reported from Angola in Africa [70], a region also previously considered free from JEV. In this case, JEV infection was believed to be locally acquired, although the potential reservoir was never confirmed, and models suggest that conditions in Africa may not be completely suitable for JEV transmission, even though competent vectors are present and both rice and pig production are increasing [71]. However, the geographical expansion of the invasive mosquito vector *An. stephensi* throughout Africa, which is fueling an increasing number of malaria outbreaks [72], highlights that mosquito population dynamics are continually evolving, and emphasizes the need for preparedness for vector-borne pathogens such as JEV.

There are several routes through which JEV could potentially be introduced to a new area, including the introduction of a viremic pig (or infected pig semen), a viremic bird, or infected mosquitoes. Along with increased global travel and the movement of people or animals, migratory birds are also thought to contribute to the geographical expansion of JEV and would therefore constitute a potential route of JEV incursion into Europe [4], as seen previously for another orthoflavivirus, WNV [73]. The distribution of JEV was previously attributed to climate (i.e., rainfall and temperature), but it has recently been emphasized that the incidence of disease in endemic areas in Asia is more likely influenced by changes in land use and land cover, for example, rice farming, cultivated land, and pig production [59, 74]. In Asia, the pig industry is increasing continuously [75], and paddy field surfaces have extended over the past 60 years [76]. Irrigated paddy fields attract wading birds and provide long-term *Culex* spp. breeding sites; hence, JEV transmission becomes less dependent on rainfall [59]. Due to these human-mediated changes, there has been an increase in both the vector, bird, and pig populations increasing the risk and magnitude of JEV circulation [59], and similar changes in Europe may increase the risk of JEV establishment in the event of an incursion. Overall, the transmission of vector-borne diseases, including JEV, is complex and multilayered, and changes cannot be attributed to climate alone [59, 77].

The current challenges to JEV diagnosis suggest that preparedness for a JEV outbreak requires a multifaceted approach. For institutes that have not previously worked with JEV, an initial challenge is adaptation to working with a new pathogen, including establishment of capability for high containment, along with the associated training, licensing and biosafety/biosecurity implications. Another potential challenge is the lack of appropriate clinical samples available for assay development, including limitations to the diversity of species for available samples. While JEV is currently not endemically detected in Europe, this will remain an ongoing challenge to any European laboratory that needs to validate JEV diagnostic assays, with samples only available through international collaborations or liaison with a WOAH Reference Laboratory. Additionally, there can be ethical considerations regarding the secondary use of human diagnostic specimens for test development, where informed consent may need to be obtained [78]. Similarly, the availability of appropriate virus strains may impact assay development, which again would need to be addressed through collaboration, although viruses are now more readily available through the European Virus Archive global (EVA global) (https://www.european-virus-archive.com). However, constraints on use, both for commercial purposes and the duration of use, can become additional hurdles. The concept and the importance of shared centralized sample storage have been discussed in the context of diagnostic preparedness for other disease outbreaks [79], where access to well characterized specimen repositories could be crucial not only for developing new diagnostic tools but for the development of more specific tests encompassing the range of JEV genotypes. Moreover, it would enhance surveillance activities and improve disease preparedness before an outbreak [79]. The majority of emerging human disease outbreaks are caused by zoonotic pathogens [80, 81]; therefore, future work on not just JEV preparedness, but preparedness for other zoonotic virus outbreaks, will need to address these limitations to plan appropriate interventions in the scientific research and development sector. Nonetheless, the limitations highlight the importance and need for collaborative and information sharing efforts [82] as exemplified by this project.

To support preparedness through establishment of robust pipelines for detection of JEV infection, implementation of appropriate prevention and control measures after initial detection of a virus incursion should also be considered. These measures could be crucial in limiting virus spread in the event of an outbreak and include the use of vaccines and potentially establishment of vaccination programs. There are several efficacious and safe vaccines against JEV currently in use, including a live attenuated vaccine, a live recombinant vaccine, and inactivated Vero cell-derived vaccines, and several new vaccine candidates are in development [1, 83, 84]. The majority of endemic countries in Asia have well-established human vaccination programs [1], however, shifts in the prevalence of predominant genotypes for a region must be taken into consideration during vaccine development [68]. Additional measures could include public health campaigns to encourage the use of mosquito repellents, long-sleeved clothes, and vaporizers [1]. However, in the event of clinical disease, there remain no licensed therapeutic agents available specifically for JE [83, 84], so treatment relies upon supportive therapy, stabilization, and relief of symptoms [1, 84]. Research in this area has identified several potential antiviral candidates; however, further research and development are needed to take them forward for evaluation in clinical trials [83, 85]. Therefore, a combined approach in establishing validated detection methods along with developing prevention and control strategies is the key to ensuring true preparedness.

## 4. Conclusions

There are several mosquito species present in Europe that are considered competent vectors for JEV. Some *Culex* species are already established throughout Europe, while several invasive *Aedes* species are becoming more common through northward geographical spread due to climate change. However, an outbreak of JEV has not occurred yet, which suggests that one or multiple (unknown) factor(s) are still lacking for a sustained transmission. The number of regions in Europe affected by outbreaks of the related orthoflavivirus WNV continues to increase [86], indicating that arboviruses have the potential to have a significant impact on both human and animal health. As multiple competent vector species of mosquito are present and expanding in Europe, knowledge gaps on competent host distributions, including the possible role of wild boar, pig-to-pig transmission, and temperature-dependent vector competence of relevant local mosquito species, should be addressed. Through this unique collaboration, we have facilitated the transparent sharing and alignment of diagnostic protocols for JEV in several European countries, to support preparedness for detection of an incursion of JEV into Europe.

## Figures and Tables

**Table 1:** Molecular protocols for the detection of JEV RNA.

Primer reference	JEV genome target	Assay type	Amplicon size (bp)
Pyke et al. [32]	NS5-3′ UTR	JEV-specific	62
Shirato et al. [33]	Capsid	JEV-specific	75
Shao et al. [34]	NS1^a^	JEV-specific	63
Yang et al. [35]	3′ UTR	JEV-specific	146
Johnson et al. [36]	NS5	Pan-orthoflavivirus	203

^a^Using the universal primer set.

**Table 2:** Detection of antibodies against JEV in sera from JEV-infected pigs, using commercially available ELISAs.

Sample ID	Reciprocal HI titer	ID Screen Flavivirus cELISA		ID Screen West Nile IgM ELISA	
		Result	S/N (%)^a^	Result	S/P (%)^b^
JEV 1	640	+	5.1	Negative	−235.8
JEV 2	640	+	9.0	Negative	−83.1
JEV 3	160	+	7.6	Negative	−0.1
JEV 4	160	+	33.1	Negative	−1.9
JEV 5	80	+	19.2	Negative	−3.6
JEV 6	80	+	21.6	Negative	−1.1
JEV 7	40	+	9.8	Negative	−4.2
JEV 8	<10	Negative	71.2	Negative	−4.8
JEV 9	<10	Negative	76.6	Negative	−4.3
JEV 10	<10	Negative	88.7	Negative	1.1
JEV 11	<10	Negative	75.4	Negative	−38.5
JEV 12	<10	Negative	70.1	Negative	−2.6

Abbreviations: HI, hemagglutination inhibition; nd, not determined; OD optical density.

^a^S/N (%) is defined as (OD sample/OD negative control) × 100, where positive samples give an S/N ≤40% and negative samples give S/N >50%.

^b^S/P (%) is defined as ([net OD sample − net OD negative control]/[net OD positive control − net OD negative control]) × 100, where net OD refers to (OD of wells with WNV antigen) − (OD of wells with dilution buffer). Positive samples give an S/P ≥45%, and negative samples give an S/P of ≤35%.

**Table 3:** Serological results for JEV samples included in the EURL equine encephalitis proficiency panel for 2023, determined at each participating institute.

Institute	Protocol	EURL Proficiency Panel sample		
		JEV 20 dpi	JEV 58 dpi	JEV 20 dpi
		(undiluted)	(1/2 dilution)	(1/4 dilution)
*ANSES* (*EURL*) *expected result*	ID Screen Flavivirus cELISA	Positive	Positive	Positive—doubtful
	VNT	1:20	1:40	1:10 or negative

WBVR	ID Screen Flavivirus cELISA	Positive	Positive	Positive
	ID Screen WNV IgM ELISA	Negative	Negative	Negative

SSI	ID Screen Flavivirus cELISA	Positive	Positive	Positive
	ID Screen WNV IgM ELISA	Negative	Negative	Negative

ANSES	ID Screen Flavivirus cELISA	Positive	Positive	Positive—doubtful
	VNT	1:20	1:40	Negative

APHA	ID Screen Flavivirus cELISA	Positive	Positive	Positive
	PRNT_50_	1:10	1:40	Negative

*Note:* The full serological report for the 2023 EURL Proficiency Panel is available on demand from adm.eurl-equinediseases@anses.fr, highlighting the alignment between participating laboratories. Samples comprised of sera from horses experimentally infected with the Tottori strain of JEV.

Abbreviation: dpi, days postinfection.

**Table 4:** Measured Ct values of the JEV samples included in the EURL equine encephalitis proficiency panel for 2023, determined at each participating institute.

Institute	Molecular assay	EURL proficiency panel sample		
		JEV GI Tottori, 1/10 (6.3 × 10^4^ PFU/mL)	JEV GIII Nakayama, 1/100 (5.1 × 10^3^ PFU/mL)	JEV GI Tottori, 1/1000 (6.3 × 10^2^ PFU/mL)
ANSES (EURL) expected Ct value		25.6	31.3	32.6

WBVR	Shao et al. [34]—universal primers	**19.0**	**23.1**	**25.8**
	Pyke et al. [32]	**18.8**	36.8	**26.0**

SSI	Shirato et al. [33]	**25.0**	**27.5**	**31.3**
	Yang et al. [35]	**20.5**	**23.9**	**26.4**

ANSES	Yang et al. [35]	**21.8**	**27.9**	33.2

APHA	Pyke et al. [32]	**20.6**	**30.7**	**27.8**
	Johnson et al. [36]*⁣*^*∗*^	**25.4**	**29.1**	34.7

SVA	Shao et al. [34]—universal primers	26.7	**28.3**	33.4
	Shao et al. [34]—GI primers	27.7	nd	34.6
	Shao et al. [34]—GIII primers	nd	**27.7**	nd

*Note:* Ct values in bold denote those below the expected Ct values defined by the EURL.

Abbreviation: nd, not done.

*⁣* ^*∗*^Pan-orthoflavivirus RT-PCR.

**Table 5:** Assessment of amplicon-based MinION protocols for WGS of JEV Nakayama (GIII) and JEV Tottori (GI).

Primer set	JEV strain	Total reads	Reads after QC	Cleaned aligned reads	Final reads (% of total reads)	Final reads (% of cleaned reads)	Consensus sequence recovery
JEV-GI (SVA)	Nakayama (GIII)	254,501	73,054	66,644	26	91	No
JEV-GI (ANSES)		137,858	19,435	17,611	13	91	No
JEV-GIII (SVA)		177,062	44,412	44,098	25	99	Yes
JEV-GIII (ANSES)		196,672	44,396	44,033	22	99	Yes

JEV-GI (SVA)	Tottori (GI)	155,212	30,888	30,627	20	99	Yes
JEV-GI (ANSES)		95,371	13,842	13,581	14	98	Yes
JEV-GIII (SVA)		111,604	12,994	11,917	11	92	No
JEV-GIII (ANSES)		123,548	22,536	21,128	17	94	No

**Table 6:** Shotgun sequencing of JEV Nakayama (GIII) and JEV Tottori (GI) (EURL proficiency samples).

EURL Proficiency Panel sample	Expected Ct (EURL)	Raw sequence reads	Mapped sequence reads^a^ (GI-Tottori)	Mapped sequence reads^b^ (GIII-Nakayama)
JEV Tottori (GI), 1/10 (6.3 × 10^4^ PFU/mL)	25.6	3,470,480	860	2
		2,583,886	675	2

JEV Nakayama (GIII), 1/100 (5.1 × 10^3^ PFU/mL)	31.3	2,363,300	0	58
		3,163,460	3	69

JEV Tottori (GI), 1/1000 (6.3 × 10^2^ PFU/mL)	32.6	3,037,448	9	0
		2,774,226	2	0

^a^Japanese encephalitis virus strain: JEV/eq/Tottori/2003, GenBank accession AB594829.

^b^Japanese encephalitis virus strain Nakayama/MY/2009/P578662, GenBank accession HE861351.

## Data Availability

The data that support the findings of this study are available from the corresponding author upon reasonable request.

## Supporting Information

Supporting Information 1 Table S1: Molecular protocols in use at partner institutes. N/A, not applicable.

Supporting Information 2 Table S2: Primer design for amplicon-based sequencing of JEV genotypes I and III, using the Primal Scheme design tool. Reference genomes were MZ540901 and EF571853 for genotypes I and III, respectively. bp, base pairs.

Supporting Information 3 Table S3: Number of JEV whole-genome sequences per species group available on NCBI GenBank (accessed February 23, 2023).

Supporting Information 4 Table S4: Primer sets for amplicon-based WGS of JEV genotype I and genotype III (designed by SVA).
