# Supplementary material for: European Preparedness for Japanese Encephalitis Virus Through Alignment of Animal Health Laboratory Diagnosis
Source: Transbound Emerg Dis. 2025 Jun 10;2025:5516160. doi: 10.1155/tbed/5516160 (PMC12173556; doi:10.1155/tbed/5516160)
Supplement: Supporting Information 1 — Table S1: Molecular protocols in use at partner institutes. N/A, not applicable. [file 5516160.f1.docx]

**Supplementary Table S1:** Molecular protocols in use at partner institutes. N/A, not applicable.

| **Assay reference** | **Sense primer** | **Antisense primer** | **Probe** |
| --- | --- | --- | --- |
| Pyke et al., 2004 | ATC TGG TGY GGY AGT CTC A | CGC GTA GAT GTT CTC AGC CC | [FAM]-CGG AAC GCG ATC CAG GGC AA-[TAMRA] |
| Shirato et al., 2005 | AGA ACG GAA GAY AAC CAT GAC TAA A | CCG CGT TTC AGC ATA TTG AT | [FAM]-ACC AGG AGG GCC CGG-[BHQ1] |
| Shao et al., 2018* | GCC ACC CAG GAG GTC CTT | CCC CAA AAC CGC AGG AAT | [FAM]-CAA GAG GTG GAC GGC C-[MGB] |
| Yang et al., 2004 | GGT GTA AGG ACT AGA GGT TAG AGG | ATT CCC AGG TGT CAA TAT GCT GTT | [FAM]-CCC GTG GAA ACA ACA TCA TGC GGC-[TAMRA] |
| Johnson et al., 2010 | GCM ATH TGG TWC ATG TGG | GTR TCC CAK CCD GCN GTR TC | N/A |

*Using the universal primer set
