# Supplementary material for: European Preparedness for Japanese Encephalitis Virus Through Alignment of Animal Health Laboratory Diagnosis
Source: Transbound Emerg Dis. 2025 Jun 10;2025:5516160. doi: 10.1155/tbed/5516160 (PMC12173556; doi:10.1155/tbed/5516160)
Supplement: Supporting Information 2 — Table S2: Primer design for amplicon-based sequencing of JEV genotypes I and III, using the Primal Scheme design tool. Reference genomes were MZ540901 and EF571853 for genotypes I and III, respectively. bp, base pairs. [file 5516160.f2.docx]

**Supplementary Table S2:** Primer design for amplicon-based sequencing of JEV genotypes I and III, using the Primal Scheme design tool. Reference genomes were MZ540901 and EF571853 for genotypes I and III respectively. bp, base pairs.

| **JEV genotype** | **Primer set** | **No. of primer pairs** | **Amplicon size**  **(bp)** | **Coverage against reference genome**  **(%)** | **5’ end not covered (bp)** | **3’ end not covered**  **(bp)** |
| --- | --- | --- | --- | --- | --- | --- |
| I | JEV-GI (SVA) | 35 | 400 | 99.8 | 11 | 14 |
|  | JEV-GI (ANSES) | 38 | 400 | 96.2 | 133 | 283 |
| III | JEV-GIII (SVA) | 35 | 400 | 99.8 | 8 | 11 |
|  | JEV-GIII (ANSES) | 37 | 400 | 97.9 | 124 | 109 |
