# Supplementary material for: European Preparedness for Japanese Encephalitis Virus Through Alignment of Animal Health Laboratory Diagnosis
Source: Transbound Emerg Dis. 2025 Jun 10;2025:5516160. doi: 10.1155/tbed/5516160 (PMC12173556; doi:10.1155/tbed/5516160)
Supplement: Supporting Information 3 — Table S3: Number of JEV whole-genome sequences per species group available on NCBI GenBank (accessed February 23, 2023). [file 5516160.f3.docx]

**Supplementary Table S3:** Number of JEV whole genome sequences per species group available on NCBI GenBank (accessed 23 February 2023).

| **Host species** | **Number of JEV whole genome sequences** |
| --- | --- |
| *Culex* spp. mosquito species | 79 |
| Porcine | 68 |
| Unspecified mosquito species | 33 |
| Human | 22 |
| Bovine | 4 |
| Other insects | 4 |
| Seal | 3 |
| Bat | 3 |
| Equine | 2 |
| Not given | 189 |
| **Total** | **407** |
